# Dr. Walter Cary

**Published:** 1881-11

**Authors:** 


### DR. WALTER CARY.

This number of the JOURNAL seems destined to be a bearer of evil tidings; for scarcely have the last words been written in regard to Dr. White, than a cablegram brings the sad intelligence of the sudden demise of Dr. Walter Cary at Marseilles, France, and Buffalo is again called to mourn one of her prominent men. It is to be regretted that brevity of time affords little opportunity to do justice to this occasion; but short as the notice must be, we cannot allow this number to go to press without paying a slight tribute to one so identified with all the interests of our city, as well as closely associated with its medical profession, though not an active member. Dr. Cary was born about the year 1812, and was between 60 and 70 years old at the time of his death; but, like Dr. White, his vigorous constitution and elastic temperament kept him youthful, in spite of the stride of time, until a few months since, when failing health cast its shadow over his life. He graduated from the University of Pennsylvania in 1843, evincing at that date, talents and characteristics which promised a more than ordinary career, and the easy circumstances of his father—the late Trumbull Cary, of Batavia—enabled him to supplement his studies with a desirable experience of some years in the hospitals on the Continent. In 1845 he associated himself with the late Dr. Charles Winne, entering into practice in Buffalo under the most favorable auspices, and gaining an enviable reputation soon after, by the zeal and skill displayed during the cholera epidemic, which for a second time made havoc in our city. Professional duties had, however, less claim on him than other more personal interests. Though frequently called in consultation, Dr. Cary gradually withdrew from the practice of medicine, and we can now but deplore that other attractions should have lost to our science his well-known energies. The remainder of his life was spent in a pleasant and large-hearted enjoyment of the many blessings prosperity had laid at his feet. His genial, social qualities brought him hosts of friends, both at home and abroad; and to the hundreds who have partaken of his open-handed hospitality the recent news carried unfeigned sorrow. We have not time, at present, to speak of the more sterling qualities and the unblemished integrity of the deceased, indeed he was too widely known in this vicinity to need an elaborate eulogy; but our sympathy—in the truest sense of the word—is extended to the large family circle which has lost a head, as Buffalo also has lost a noted citizen.

